# Transcriptional Activity of the *FUT1* Gene Promoter Region in Pigs

**DOI:** 10.3390/ijms141224126

**Published:** 2013-12-11

**Authors:** Chen Zi, Zhengchang Wu, Jing Wang, Yongjiu Huo, Guoqiang Zhu, Shenglong Wu, Wenbin Bao

**Affiliations:** 1 Key Laboratory for Animal Genetics, Breeding, Reproduction and Molecular Design, College of Animal Science and Technology, Yangzhou University, Yangzhou 225009, Jiangsu, China; E-Mails: zchandy@163.com (C.Z.); wuzhengchang@126.com (Z.W.); jinghost@163.com (J.W.); huoyj@126.com (Y.H.); 2 College of Veterinary Medicine, Yangzhou University, Yangzhou 225009, Jiangsu, China; E-Mail: yzgqzhu@yzu.edu.cn

**Keywords:** pig, *FUT1* gene, promoter, transcriptional activity, luciferase

## Abstract

This study aims to provide a theoretical basis on the regulatory mechanism of the α-l,2-fucosyltransferase (*FUT1*) gene in pigs by analyzing the transcriptional activity of its promoter region. On the basis of the previously obtained promoter sequence, primers upstream and downstream of the gene were designed using the restriction endonucleases *Kpn*I and *Hind*III respectively, and the recombinant plasmids of the pGL3-promoter were constructed by inserting promoter sequences with partially missing regions. The resultant mutants were observed by transient transfection assay into HEK293 cells, and the transcriptional activity of the promoter region was determined by luciferase activity. The 5′-flanking region of the *FUT1* gene (−1150 to +50 bp) exhibited promoter activity. The −1150-bp to −849-bp region showed negative regulation of the gene. The recombinant plasmid pGL3-898 showed the strongest luciferase activity, and the activity showed a decreasing trend when the deleted region was increased. Recombinant plasmids were successfully constructed, verified, and the positive and negative regulation areas and core promoter region were detected, providing a deeper insight into the transcriptional regulatory mechanism of the *FUT1* gene.

## 1. Introduction

Porcine post-weaning diarrhea (PWD) and porcine edema disease (ED) are two diseases caused by enterotoxigenic *Escherichia coli* F18 (ETEC F18). These diseases are responsible for tremendous damage and economic losses in the pig industry [1]. Some molecular genetic studies by both national and international researchers have investigated the factors determining immunological resistance of pigs to ETEC F18. The α-l,2-fucosyltransferase (*FUT1*) gene can affect the expression of the ETEC F18 receptor gene *ECF18R* and an M307 mutant locus in the *FUT1* gene was a genetic marker for selecting new pig cell lines resistant to ETEC F18 [2]. Meijerink *et al*. [3] found a G/A mutation at the M307 locus of the *FUT1* gene where G was predominant over A. In other words, pigs with the AA genotype exhibited ETEC F18 resistance while pigs with the GG genotype were susceptible. However, other studies that analyzed the polymorphism at the M307 locus discovered that over 20 Chinese native pig breeds had only the GG genotype, and the distribution of these breeds in China was extremely skewed [4–7]. These findings suggested that differences other than the coding region in the *FUT1* gene may exist between native Chinese and commercial western pig breeds. Therefore, further studies are required to verify and validate the regulatory mechanism of ETEC F18 resistance in the *FUT1* gene.

A promoter region consists of transcriptional start sites (TSSs), and the transcription factor recognizes and specifically binds to *cis*-acting elements in the promoter region. Meanwhile, DNA methylation and base mutations in the promoter region cause changes of genetic expression. Therefore, it is necessary and significant to study the regulatory mechanism of the promoter region in the *FUT1* gene. Research on the upstream regulatory mechanism of the *FUT1* gene could help explain the genetic difference between native Chinese and commercial western pig breeds and provide certain theoretical foundation for breeding pigs with ETEC F18 resistance.

Currently, there is no information about the *FUT1* promoter region sequences in existing authoritative databases. Moreover, there remains a gap in the regulatory mechanism of ETEC F18 resistance in the *FUT1* promoter region; therefore, it is difficult to sequentially probe into the *FUT1* promoter region. In our study, we obtained the sequences of the *FUT1* promoter region from previous chromosome walking studies [8] and reconstructed the fragment-deleted vectors. The transcriptional activity of this gene was determined by measuring the expression level of luciferase. Based on the research on transcriptional activity of sequences in promoter region, the key regulatory region was verified and provided a theoretical basis for realizing the *FUT1* gene promoter function.

## 2. Results and Discussion

### 2.1. PCR Amplification

**Figure 1.**
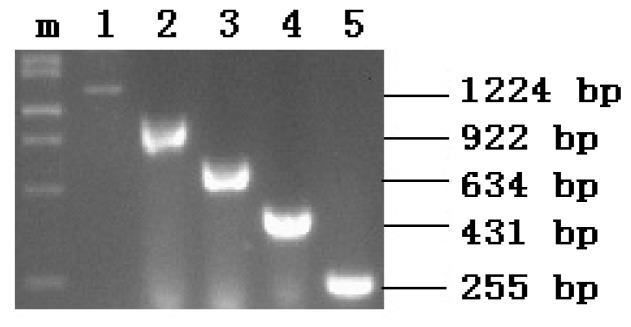
Identification of the PCR products by electrophoresis in 1% agarose gel. **m**: DNA marker; **1**, **2**, **3**, **4**, and **5** denote the PCR products.

PCR products were verified by electrophoresis in 1% agarose gels stained with ethidium bromide. Five clear DNA bands were observed at 1224, 922, 634, 431, and 255 bp; this was in agreement with the predicted amplified fragment sizes. The results indicated successful recovery of the deleted DNA fragments. The electrophoresis map is shown in Figure 1.

### 2.2. Recombinant Plasmid Enzyme Identification

**Figure 2.**
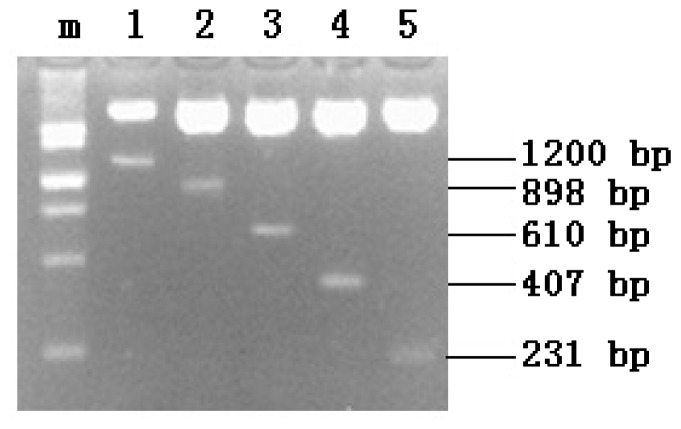
Identification of the recombinant plasmids by double enzyme digestion. **m**: DNA marker; **1**, **2**, **3**, **4**, and **5** denote the double enzyme digestion of pGL3-1200, pGL3-898, pGL3-610, pGL3-407, and pGL3-231, respectively.

The recombinant plasmids with inserts were digested with *Kpn*I and *Hind*III, resulting in five fragments with inserts (Figure 2). Further sequencing analysis revealed that the sequences of the inserts were consistent with the sequences previously determined by chromosome walking, indicating that the directional construction of promoter serial deletion plasmids of the *FUT1* gene, namely, pGL3-1200, pGL3-898, pGL3-610, pGL3-407 and pGL3-231, was successful.

### 2.3. Luciferase Activity Analysis of Recombinant Plasmids in the Deleted Fragments of Pig *FUT1* Gene Promoter

**Figure 3.**
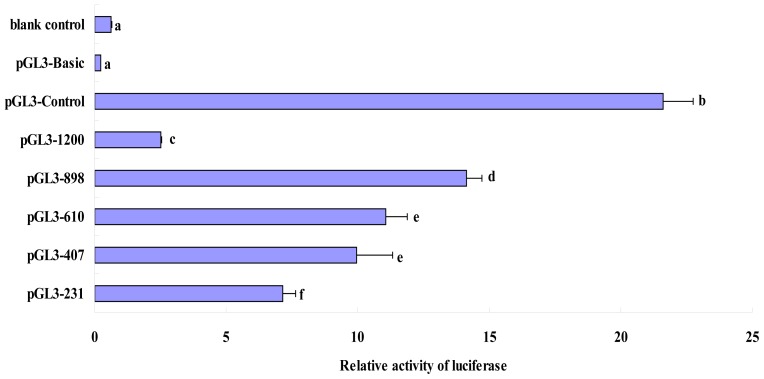
Luciferase activity analysis of recombinant plasmids in HEK293 cells. Different letters indicate significant differences (*p* < 0.01).

Differences in the promoter activity between pGL3-610 and pGL3-407 were not significant; however, the differences in other groups were significant (Figure 3). Of these, promoter activity of pGL3-898 was the highest (14.124 ± 0.605) and that of pGL3-1200 was the lowest (2.509 ± 0.035), indicating that the deletion in the pGL3-898 plasmid included a key *cis*-acting element that functioned in negative regulation. The promoter activities decreased in the order pGL3-610 (11.050 ± 0.817), pGL3-407 (9.968 ± 1.349), and pGL3-231 (7.132 ± 0.520), indicating that the deleted regions played a role in positive regulation (Figure 3).

Sequence mutations in the promoter region have been associated with diseases as well as gene expression levels. Studies have found that a nucleotide mutation in the interleukin-4 (*IL-4*) promoter region was related to the forced expiratory volume per second (FEV1) in asthmatic patients [9], and a nucleotide mutation in the promoter region of the β-globin gene was found to be associated with anemia [10]. Studies have shown that methylation in the promoter regions of the human *p15* and *p16* genes was associated with cervical cancer and significantly inhibited transcription of the *p16* gene [11]. In view of the importance of the promoter, studies have highlighted the functions and features of specific sequences in the regulatory region and their involvement in gene expression. The promoter activity of a transfected fragment is determined by reporting the fluorescence value of the gene by using a Dual-Luciferase Reporter Assay System and transient transfection technique. The core promoter region and major transcription factor-binding regions are thus determined. The promoter sequences in sequence-deleted gene *PIT-1* of goose into a luciferase expression vector, and determined the positive and negative regulatory regions and the core promoter region by using transient transfection [12]. Similarly, the promoter activity of the TP526 sequence in the upstream regulatory region of pig *THRSP* was tested using the above technique [13]. The inserted 304-bp sequence in the 5′-flanking region of the pig *IDH3β* gene could improve the transcriptional levels [14]. The *FUT1* gene in swine is closely related to diarrhea and edema in piglets and the coding regions of interest in this gene have been comprehensively researched. It is necessary to verify and analyze the functions of the promoter regions. Based on searching the important region in transcriptional regulation, the possible nucleotide differences and susceptibility to disease should be validated and verified in order to determine the genetic differences between native Chinese and commercial western pig breeds.

In this experiment, 1200-bp (−1150-bp to +50-bp) sequences of the *FUT1* gene were harvested according to previously reported results of chromosome walking. The CpG island in the upstream regulatory region was predicted using online software CpGPlot. The predictive findings indicated that the regions of approximately −950-bp to −280-bp were rich in CpG islands, indicating that methylation may occur in the region at the 5′-flanking region and regulate the transcriptional level of the gene by affecting the binding of transcriptional factors.

According to our results, the HEK293 cells transfected with the pGL3-898 vector showed the maximum luciferase activity, which was significantly higher than that of pGL3-1200 and the other sequence-deleted recombinant vectors. Compared with pGL3-1200 vector, the pGL3-898 is short of the −1150-bp to −849-bp region. According to the results, pGL3-898 vector showed the maximum luciferase activity, precisely because the deficiency of the −1150-bp to −849-bp region. When the −1150-bp to −849-bp region was deleted in the promoter sequence, the deleted sequence exhibited negative regulatory effects on the transcription of the *FUT1* gene. The online software TFSEARCH (http://mbs.cbrc.jp/research/db/TFSEARCH.html) was used for prediction, and the results revealed that the partially deleted fragments existed in transcriptional elements such as AML-1a, GATA-3, GATA-2, Nkx-2, HFH-2, CHOP-C, Tst-1, SRY and HNF-3b and the predictive value of Nkx-2 was indicated to be 100. The deleted sequence showed a negative regulatory effect although it contained abundant *cis*-acting elements and transcription factor-binding loci, suggesting that the element in the predicted locus may not play a key role in transcription regulation of the *FUT1* gene.

The luciferase activity of pGL3-610 and pGL3-407 did not significantly differ, and their activities were significantly lower than that of pGL3-898. The −848-bp to −561-bp region was deleted in pGL3-610, whereas the −560-bp to −358-bp region was deleted in pGL3-407. MZF1, v-Myb, Elk-1, c-Ets-1, CDP CR, GATA-1, and MyoD were predicted to exist in the −848-bp to −561-bp region; two SP1, CP2, two MZF1, two NRF-2, c-Ets-1, and GATA-1 binding sites were predicted to exist in the −560-bp to −358-bp region. The −357-bp to −182-bp region was deleted in pGL3-231 and was predicted to contain the binding sites for GATA-1, deltaE, two SP1, and MZF1.

The promoter region of pGL3-231 contained the −181-bp to +50-bp region of the candidate sequence. According to luciferase activity of the recombinant vector, we predicted the region contained SP1, Ik-2, GATA-1, c-Ets-1, MZF1, GATA-2, Ik-2, Elk-1, and CdxA. The RNA polymerase in eukaryotes generally combined with the sequence 30 bp upstream from the transcription start site. Such a vector contains a key sequence that is recognized by RNA polymerase and initiates the transcription so that the vector has a relatively higher transcriptional activity. Predictive findings showed that there was no TATA box in the promoter region, indicating that the *FUT1* gene promoter region may not contain the typical TATA box that is recognized by RNA polymerase II. Therefore, not all promoter regions in each gene contain the special structure [15,16].

We used online TRANSFAC (http://www-bimas.cit.nih.gov/molbio/signal/) to predict that two CATCTG sequences exist in promoter region of the *FUT1* gene. Studies have found that the core sequence of the 3′-enhancer is a trans-acting factor-binding motif [17]. The pGL3-610 was found to contain two such sequences at the −371 and −353 loci, while the pGL3-407 vector contained only one sequence at the −353 locus. The transcriptional activities of the pGL3-610 and pGL3-407 vectors did not significantly differ, but they were significantly higher than that of the pGL3-231 recombinant vector. This finding indicated that the two sequences positively regulated the *FUT1* gene to some extent, and that the sequence of CATCTG at the −353 locus may be an indispensable part of transcription regulation.

After determining the key regulatory region of the *FUT1* gene promoter, we will further investigate the different effects of polymorphic and methylation loci in critical regulatory regions of *FUT1* promoter on the regulation of gene expression. Moreover, the genetic effect of functional mutant loci in key promoter regulatory regions in native Chinese pig breeds that result in *E. coli* F18 resistance will be validated and verified. The findings of our study provide a theoretical basis for the resistance of native Chinese pig breeds to *E. coli* F18.

## 3. Experimental Section

### 3.1. Experimental Materials

Healthy Sutai pigs (35 days old) were obtained from the Sutai Pig Breeding Center in the city of Suzhou, Jiangsu Province. Ear tissue samples were collected from the Sutai pigs, and DNA was extracted from each sample by using a routine phenol-chloroform method. The pGL3-basic vector and AccuPrime Pfx polymerase were purchases from Invitrogen (Carlsbad, CA, USA). *Kpn*I and *Hind*III endonucleases were obtained from MBI (Lansing, MI, USA). DNA ligase was purchased from New England Biolabs (Beverly, MA, USA). The Dual-Luciferase Reporter Assay System was purchased from Promega (Madison, WI, USA). *E. coli* strain DH5α and human embryonic kidney 293 (HEK293) cells were obtained from ATCC (Rockville, MD, USA).

### 3.2. Primer Design of Pig *FUT1* Gene Promoter

On the basis of the 1200-bp fragment (−1150 to +50 bp) of the *FUT1* gene sequenced by chromosome walking, we designed five primer pairs of different lengths for PCR: 1200, 898, 610, 407, and 231 bp. 6 base pairs of protective base (5′-ATCATC-3′), *Kpn*I and *Hind*III sites were added upstream and downstream of the 5′-end of the primer pairs, respectively. The primer sequences and annealing temperature are shown in Table 1.

### 3.3. Preparation of Deleted Fragments of Pig *FUT1* Gene Promoter

The DNA was amplified in a 50-μL reaction mixture containing 5 μL of 10× AccuPrime Pfx Reaction Mix, 5 μL of 10× enhancer, 1 μL (10 μmol/L) of each primer, 1 μL of DNA template, and 0.5 μL of AccuPrime Pfx polymerase, with sterilized distilled water to make up the final reaction volume of 50 μL. The PCR amplification protocol consisted of denaturation at 95 °C for 3 min; 28 cycles for denaturation at 95 °C for 30 s; annealing at 57–59 °C for 30 s, and extension at 68 °C for 30–90 s; followed by a final extension step at 68 °C for 10 min. The PCR products were stored at 4 °C until use for experiments. PCR products were verified by electrophoresis in 1% agarose gels and subsequent staining with ethidium bromide.

The resulting PCR fragments and the pGL3-basic plasmid were digested using *Kpn*I and *Hind*III. The 50-μL system of enzyme digestion consisted of 600 ng of PCR fragments or plasmid, 2 μL of *Kpn*I or *Hind*III, 5 μL of 10× *BamH*I buffer, and sterilized distilled water to make up the final volume of 50 μL. PCR fragments or plasmid was digested for 2 h at 37 °C using the *Kpn*I or *Hind*III restriction enzyme. The target fragment was recycled through the column, and the pGL3-basic plasmid was recycled from agarose gel.

### 3.4. Vector Construction and Verification

The ligation mixtures of each PCR fragment and the plasmid consisted of 0.5 μL of 10× ligation buffer, 3.0 μL of DNA fragments (*Kpn*I/*Hind*III), 1.0 μL of pGL3-basic plasmid (*Kpn*I/*Hind*III), and 0.5 μL of T4 DNA ligase (10 U/μL). The ligation mixtures were then used to transform *E. coli* DH5α cells. The positive recombinant products were selected on Luria-Bertani (LB) agar plates containing 100 μg/mL ampicillin, and confirmed by PCR and DNA sequencing.

The fragments of interest were recovered from the agarose gel, purified, and ligated by T4 DNA ligase, resulting in the recombinant plasmids pGL3-1200, pGL3-898, pGL3-610, pGL3-407, and pGL3-231 (named by target fragment length).

### 3.5. Luciferase Activity Assay

HEK293 cells were seeded on 12-well plates (at a density of 2 × 10^4^ cells/well) 24 h before the start of transfection. Then, 1 μg of plasmid and 25 ng of pRL-TK were diluted in 250 μL of Opti-MEM and divided into eight groups: control (no transfection), pGL3-basic (no insert), pGL3-control, pGL3-1200, pGL3-898, pGL3-610, pGL3-407, and pGL3-231. Then, Lipofectamine 2000 (10 μL) was diluted with 250 μL Opti-MEM and added to the diluted plasmid described above. The mixtures were then incubated for 20 min at room temperature. Cells were co-transfected with the mixture by incubation in antibiotic-free complete medium for 48 h. Cell lysates were obtained and the luciferase activities were determined using the Dual-Luciferase Reporter Assay System according to the manufacturer’s instructions. The relative luciferase activity (RLA) was determined by normalization with Renilla luciferase activity using a Modulus microplate multimode reader (Turner Biosystems, Sunnyvale, CA, USA).

## 4. Conclusions

In this study, the sequence-deleted fragment in the regulatory region was subcloned into the firefly luciferase plasmid pGL3-basic and transiently co-transfected into HEK293 cells with the internal reference plasmid pRL-TK containing sea pansy luciferase. The transcriptional activity of the promoter sequence of *FUT1* was determined using the Dual-Luciferase Reporter Assay System in order to explore the regulatory element and mechanism of transcription initiation in the pig *FUT1* gene. The results showed that the recombinant vectors exhibited promoter activity, although there was no typical TATA box upstream regulatory region near the transcription start area. The recombinant vector pGL3-898 showed maximum transcriptional activity and the pGL3-898 was short of −1150-bp to 849-bp region, so that the −1150-bp to −849-bp region exhibited a negative regulatory effect on transcription initiation in the upstream non-coding region of the *FUT1* gene, in spite of abundant *cis*-acting elements and transcription factor-binding loci predicted in the deleted sequence. That suggested this region should be the focus of further research using polymorphic and methylation methods. The luciferase activity showed a decreasing trend when the deleted region was increased, and there were also abundant *cis*-acting elements and transcription factor-binding loci in region of −848-bp to 50-bp, suggesting that the sequence of interest has an important positive regulatory effect in the transcription of the *FUT1* gene, and deeper research is necessary.

## Figures and Tables

**Table 1.** Primer sequences of different lengths of *FUT1* gene promoter. P is the shared reverse primer, and A, B, C, D and E respectively represents the forward primers used for producing PCR fragments with different length. The lower case letters are the recognition site of the restriction enzyme.

Name	5′-Sequences-3′	Amplification Region (bp)	Annealing Temperature (°C)
A (*Kpn*I) P (*Hind*III)	ATCATCggtaccCTGGCCTGGCTCAGTGGGTCAGGGACCCAGC ATCATCaagcttCTGAGCTCCTGGCGGGTGGCAGATTCCCAG	(−1150)–50	57
B (*Kpn*I) P (*Hind*III)	ATCATCggtaccCGGCCTTTGTTCCAGAAGCCGGCTGCAGCCCGGGCCT ATCATCaagcttCTGAGCTCCTGGCGGGTGGCAGATTCCCAG	(−848)–50	58
C (*Kpn*I) P (*Hind*III)	ATCATCggtaccTGCTGCCCCCGGGGAGGACTCGGCAGGGGGGCGGGGGG ATCATCaagcttCTGAGCTCCTGGCGGGTGGCAGATTCCCAG	(−560)–50	58
D (*Kpn*I) P (*Hind*III)	ATCATCggtaccGCTGCATCTGGCCGCTGGATCTCCGCGGCCG ATCATCaagcttCTGAGCTCCTGGCGGGTGGCAGATTCCCAG	(−357)–50	59
E (*Kpn*I) P (*Hind*III)	ATCATCggtaccGCGCCAGGGAAGGGGTGGGGCTCCGCCTCCG ATCATCaagcttCTGAGCTCCTGGCGGGTGGCAGATTCCCAG	(−181)–50	59
